# Alternative Paradigm of Selective Vagus Nerve Stimulation Tested on an Isolated Porcine Vagus Nerve

**DOI:** 10.1155/2014/310283

**Published:** 2014-02-06

**Authors:** Polona Pečlin, Janez Rozman

**Affiliations:** ITIS d.o.o. Ljubljana, Center for Implantable Technology and Sensors, Lepi Pot 11, 1000 Ljubljana, Slovenia

## Abstract

Alternative paradigm for spatial and fibre-type selective vagus nerve stimulation (VNS) was developed using realistic structural topography and tested in an isolated segment of a porcine cervical left vagus nerve (LVN). A spiral cuff (cuff) containing a matrix of ninety-nine electrodes was developed for selective VNS. A quasitrapezoidal stimulating pulse (stimulus) was applied to the LVN via an appointed group of three electrodes (triplet). The triplet and stimulus were configured to predominantly stimulate the B-fibres, minimizing stimulation of the A-fibres and by-passing the stimulation of the C-fibres. To assess which fibres made the most probable contribution to the neural response (NR) during selective VNS, the distribution of conduction velocity (CV) within the LVN was considered. Experimental testing of the paradigm showed the existence of certain parameters and waveforms of the stimulus, for which the contribution of the A-fibres to the NR was slightly reduced and that of the B-fibres was slightly enlarged. The cuff provided satisfactory fascicle discrimination in selective VNS as well as satisfactory fascicle discrimination during NR recording. However, in the present stage of development, fibre-type VNS remained rather limited.

## 1. Introduction

In recent decades, considerable scientific and technological efforts have been devoted to the development of neuroprostheses that interface the human autonomic nervous system with electronic implantable devices. Particular attention has been paid to VNS techniques that are to be used to treat, among others, a number of nervous system disorders, neuropsychiatric disorders, eating disorders, sleep disorders, cardiac disorders, endocrine disorders, and pain [1–5].

In the current research and clinical practice, however, the stimulation of LVN is more frequently used than the stimulation of right vagus nerve. In adults, the right vagus nerve innervates the sinoatrial (SA) node, the atrial muscle and, to a much lesser degree, the atrioventricular (AV) node, whereas the LVN innervates the SA node and atrial muscle to a lesser degree than it innervates the AV node [6].

In many heart diseases, for instance, such as hypertension and congestive heart failure, cardiac vagal activity is diminished and unresponsive. Numerous studies have therefore proposed VNS also as a method for treating various heart conditions, including supraventricular arrhythmias, angina pectoris, congestive heart failure, atrial fibrillation, myocardial ischemia, and variant angina (spastic coronary arteries) [2, 7–12].

In addition, brain-stimulation methods are making significant inroads into psychiatric practice. For instance, VNS has also been approved as a therapy for medication-resistant depression [13, 14]. Finally, VNS is used worldwide as a non-pharmacological treatment to control seizures in epilepsy patients [15].

However, the method commonly used is in general a non-selective VNS of the LVN, which in turn causes frequent occurrence of undesirable side effects [16]. In addition, afferent VNS leads to reflex-excitation of vagal efferent activity and inhibition of sympathetic efferent activity [17].

To alleviate such problems, various selective VNS models and electrode systems that selectively stimulate certain features have been developed, that is, intermediate-diameter B-fibres in a nerve while avoiding the stimulation of A-fibres and C-fibres [18].

In the field of functional electrical stimulation, cuffs have been used as stimulation electrodes as well as electrodes for NR recording for more than 35 years [19–21]. Since then, theoretical considerations and different models have accelerated the development of cuffs [22–24].

Prosthetic devices that are controlled by cuffs recording neural activity are a reality today. However, the amount of information that can be extracted from these recordings is still limited [25].

For reliable and safe VNS using cuffs, the response of neural elements to stresses that may occur during the complex interactions taking place between the electrode and stimulated nerve, must be understood [26]. The successful long-term use of cuffs for selective VNS may be restricted by unreliable NR recordings, owing to changes in the complex impedances of the stimulating and recording electrodes [27]. Furthermore, currently there does not exist a type of multi-electrode system that adequately combines spatial and fibre-type VNS with recording.

This paper presents the hypothesis concerning both selective activation of B-fibres and selective recording of NR, wherein both stimulation and recording were performed in a single fascicle with a single-part ninety-nine-electrode cuff fitted around an isolated porcine LVN and maintained at physiological temperature in oxygenated artificial cerebrospinal fluid. One specific aim was to determine which precise parameters of the applied pulses were most effective for fibre-type VNS.

## 2. Methods

### 2.1. Model of Porcine LVN Stimulation

In this study, the porcine LVN as an animal model was adopted. The LVN is especially suited for evaluation of fiber-type selective methods because it is composed of the following three distinct groups of myelinated fibres: A*β*, hereafter referred to as A-fibres (diameter of the fibre (*D*) = 5–12 *μ*m), myelinated A*δ*-fibres, also designated as B-fibres (*D* = 1–5 *μ*m), and unmyelinated C-fibres (*D* = 0.5–2 *μ*m) [28]. The vast majority are C-fibres, whereas A- and B-fibres are in minority. A- and B-fibres in vagus nerve, however, are primarily related to innervation of cardiovascular and respiratory systems [29–38].

### 2.2. Multielectrode Cuff Design

The cuff was designed considering both published results of studies modelling the selective stimulation of peripheral nerves [21–23, 39] and a realistic structural topography of the porcine LVN [18]. It should be noted, however, that models of myelinated nerve fibers, used to investigate electrical nerve stimulation, are highly stylized abstractions of real fibers.

To define the relationship between the structural topography and the physical model, the distributions of fibre diameters and physical dimensions in three porcine LVNs were measured. For this purpose, approximately 8 cm long and 3 mm thick LVN was taken out from the midcervical neck of a pork (about 80 kg in weight) and fixated for histological examination. When fixated, three microtome sections were cut from each LVN at the following sites: at the middle of the LVN, at a distance of 14 mm left from the middle of the LVN, and at a distance of 14 mm right from the middle of the LVN.

A section cut from the middle of the LVN was considered as the site of stimulation origin, while the other two sections represented the sites of NR recording. For the presented paradigm, only microtome sections cut at the middle of the LVNs were analyzed. For the expression of myelin in microtome sections an immunohistochemical marker of neural tissue, namely, anti-S-100 protein antibody (PAP method), was used. Accordingly, great majority of the nerve fibers in the LVN were not expressed, since they were unmyelinated (C-fibres). Out of counted 500 myelinated fibres, 200 of them were of the diameter larger than 5 *μ*m (A-fibres), while 300 of them were of the diameter smaller than 5 *μ*m (B-fibres). Corresponding results of histological examinations and physical measurements in the LVNs, adopted in the paradigm, are shown in Table 1.

The cuff was produced by bonding two 0.05 mm thick silicone sheets together (BioPlexus Corporation, Ventura, CA, U.S.A.). One sheet, stretched and fixed in position, was covered with an adhesive layer (Applied Silicone Corporation, Santa Paula, CA, U.S.A.). The second, unstretched sheet was placed on top of the adhesive, and the composite was compressed to a thickness of 0.15 mm. When released, the composite curled into a spiral tube as the stretched sheet contracted to its natural length [20, 21].

**Figure 1:**
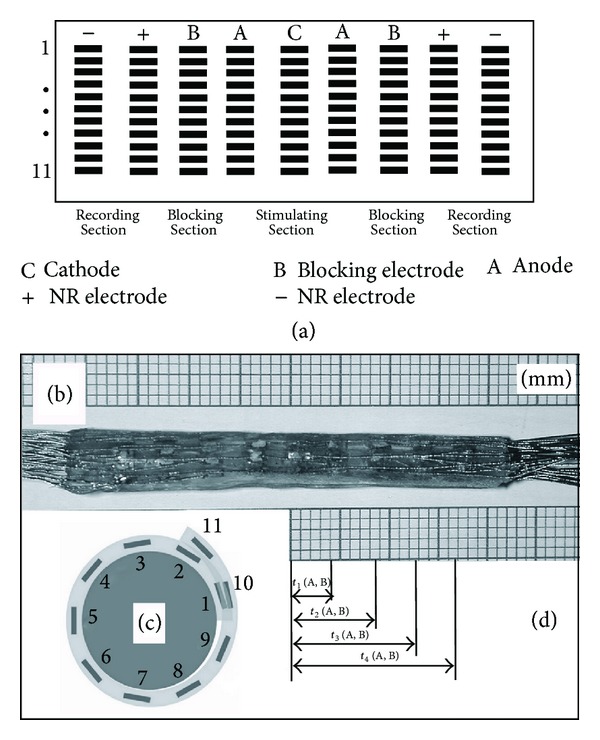
(a) Matrix of ninety-nine electrodes, (b) fabricated cuff, (c) cross-section through the cuff, and (d) distances between spiral groups of electrodes and times used in calculations of the CV in the A- and B-fibres.

Ninety-nine rectangular electrodes made of 45 *μ*m thick platinum ribbon (99.99% purity) were mounted on a third silicone sheet. These were arranged in a matrix of nine parallel groups, each containing eleven electrodes, and divided into the following sections (Figure 1(a)): stimulating section, containing eleven groups of three electrodes (triplet 1–11), positioned at the middle of the matrix; two blocking sections, having eleven electrodes each, positioned bilaterally next to the stimulating section; and two recording sections, having eleven spiral couples (couple 1–11), positioned next to the blocking sections.

Afterwards, the silicone sheet containing the matrix of electrodes was adhered to the inner side of the spiral tube. Figure 1 shows a fabricated cuff 44 mm in length (b) and schematic cross-section through the cuff (c).

### 2.3. Selective VNS

In myelinated nerve fibres, action potential (AP) is regenerated only at the nodes of Ranvier and propagate by jumping from one node to a subsequent node at rapid CV [40, 41]. One assumption made was that of an anatomical feature previously explained by Rushton [42], that is, in myelinated fibres, the internode length and the thickness of the myelin sheath are both proportional to D, and that the spike CV shows an approximately linear relationship to axon diameter throughout the entire range of myelinated fibres. Data considering the CV of different nerve fibres was adopted from the literature [43–46].

The differing properties of each individual fibre result in different AP thresholds, refractory periods, and the duration of the AP [47]. Consequently, any change in the amplitude and waveform of the NR amplitude is due to a change in the number of fibres that are firing. With increasing intensities of a stimulus, however, the number of axons firing is equivalent to the sum of all those, whose thresholds are met by a given input. The latency between the application of the stimulus and the onset of the NR is a function of the events during the depolarization and the distance between the recording site and the site of the stimulation.

A difficulty observed in previous studies of nerve stimulation is that the difference in stimulating (cathodic) intensity between the threshold excitation and the maximum recruitment of myelinated fibres is not large [48, 49]. A more selective recruitment of myelinated fibres can therefore be obtained if it is possible to exploit the difference in the threshold between the different fibre diameters. However, the conventional stimulation of nerves, using biphasic rectangular stimuli, unavoidably excites the larger nerve fibres before the smaller ones [50]. One possibility to overcome this difficulty is to introduce an exponentially decaying anodal block of nerve conduction, as described in the model of Accornero et al. [51]. Later on, it was also demonstrated by Fang and Mortimer [39] and others [52, 53] that the A-fibres could be activated and blocked at lower currents than the B-fibres. This is because the B-fibres have more nodes per unit length than the A-fibres, so they require a higher excitation voltage and thus a higher injected current to activate the AP as well as to elicit a block. With this regard it was proposed that the stimulating intensity, required for the activation AP in the A-fibres, would be slightly lower than the threshold for the B-fibre activation. Such activation would enable also the use of an intensity that is sufficiently strong to activate the B-fibres. Taking into account the above-mentioned differences in the excitation threshold and the CV of the A- and B-fibres it was assumed that the latest AP of the A-fibres arrives at an inner edge of the triplet anode at a time when the hyperpolarizing effect at the anode is still strong and should be blocked by hyperpolarization. This, in effect, produces an anodal block that prevents the propagation of the AP of the A-fibres towards the recording couple. However, the hyperpolarization should be decayed exponentially to prevent any anodal break excitation, which had previously been a problem in the stimulation with conventional rectangular pulses [39]. In addition, the intensity of the anodic phase of the stimulus had to be lower than the threshold of the anodic activation of the A-fibres in order to prevent the excitation due to the reversed polarity on the cathode during the anodic phase. According to the paradigm, an exponentially decaying part of cathodic phase of the stimulus should last until the latest AP of the A-fibres passes the outer edge of the blocking electrode. The AP in the B-fibres, however, should reach an inner edge of the triplet anode after the cathodic intensity has exponentially decayed to a point that is inefficient at blocking their conduction. The selectivity of the technique for fibres of different CV could therefore only be achieved by changing the time constant of the exponential decay and by adjusting the stimulating cathodic intensity.

**Figure 2:**
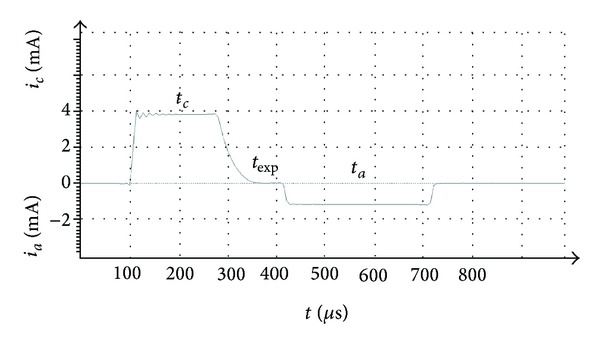
Parameters and waveform of the stimulus determined in the paradigm.

The resulting stimulus shown in Figure 2 was a current, biphasic, charge-balanced, and asymmetric pulse, composed of a precisely determined quasitrapezoidal cathodic phase with a square leading edge of intensity *i*_*c*_, a plateau of the cathodic phase with the width of *t*_*c*_, followed by an exponentially decaying phase with the width of *t*_exp⁡_ and the time constant *τ*_exp⁡_, and ended by a wide, rectangular, anodic phase with the width *t*_*a*_ and intensity of *i*_*a*_.

As a result of the cuff design, the following dimensions of the cuff were adopted:

 nominal nerve diameter: 2.5 mm,
 cuff length: 44 mm,
 electrode length: 2 mm,
 electrode width: 0.5 mm,
 circumferential separation between electrodes: 0.5 mm,
 longitudinal separation between electrodes: 2 mm.

It was presumed that the A- and B-fibres could be activated at any site on the cathode and the corresponding AP would propagate simultaneously in both directions. The population of closely spaced nerve fibres above an appointed triplet with roughly synchronous firing pattern was considered as a single pathway.

It was also assumed in the paradigm that an additional blocking electrode situated next to the triplet anode would provide an additional blocking effect of the A-fibres. In the two blocking sections of eleven electrodes, each one was galvanically connected to the neighboring triplet anode.

The efficacy of the selective VNS and recording of the NR are strongly dependent on the physical proximity of the deployed electrodes and the distance between the electrodes and the nerve fibres. To stimulate a certain group of fibres in a fascicle, while also avoiding causing injury associated with high charge density, a well-defined electrical charge should be applied to preselected locations [54]. To keep the electrode-electrolyte interface within capacitive mechanisms, the cathode was dimensioned so as not to exceed the limits for a reversible charge injection [55].

In the proposed paradigm, the view of Sunderland and Bedbrook [56], wherein fibres in fascicles extending peripherally along a nerve extensively interweave so that they converge and diverge into new and different fascicular assemblies, was adopted. For this reason, the distance between the stimulating cathode and the couple within the row of nine electrodes was fixed to be well below that at which the confluence of fibres significantly changes (Figures 1(a) and 1(b)).

Only myelinated A-fibres (CV = 30–70 m/s) and myelinated B-fibres (CV = 3–15 m/s) were considered in the paradigm. The unmyelinated C-fibres (CV = 0.5–2 m/s) however, could not be activated by the proposed stimuli, so they were not considered.

Taking into account the differences in the CV of the A- and B-fibres that could potentially contribute to the NR during the selective VNS, Table 2, describing the propagation of AP in the A- and B-fibres from the triplet cathodes to different groups of electrodes within the cuff, was compiled (Figure 1(d)). For this purpose, a previously reported calculation method, using length and latency differences between stimulated and recording sites on a LVN, was employed [57, 58].

### 2.4. Selective NR Recording

The fact that different contacts in a cuff preferentially record different pathways in the nerve, demonstrated by numerous authors, was considered [23, 59–62].

It was obvious that fibre distribution, electrode separation in the couples, and the distance between the active fibres and the recording electrode would all have major roles in determining the peak-peak NR amplitude [63].

According to the paradigm, positive NR deflection was expected to occur at the moment when the eventually nonblocked A-fibre and particularly fastest B-fibre AP reached the inner edge of a positive recording electrode, lasting until the last of the B-fibre AP passed the outer edge of a positive recording electrode. However, negative NR deflection was expected to occur at the moment when the eventually nonblocked A-fibre and fastest B-fibre AP reached the inner edge of a negative recording electrode, and lasting until the last of the B-fibre AP passed the outer edge of a negative recording electrode.

### 2.5. Preparation of Nerves

Experiments were performed in four Slovenian male Landrace pigs weighing about 150 kg each. The animals were killed according to established protocols for mature animals, sows, or boars (Penetrating Captive Bolt and immediate discharge of blood). The obtained neural tissue was treated in accordance with the approval provided by the ethics committee at the Veterinary Administration, Ministry of Agriculture, Forestry and Food, Republic of Slovenia (number: 34401-27/2010/3).

**Figure 3:**
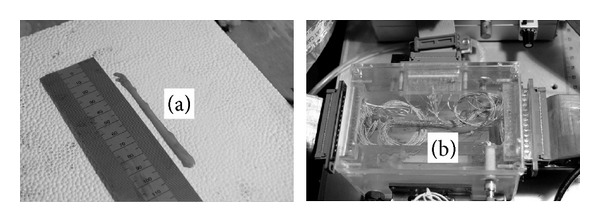
(a) Isolated LVN and (b) LVN within the cuff mounted into an experimental chamber.

The LVN was removed from the approximately 10 cm long porcine midcervical neck, carefully freed from excessive fat tissue and shortened to about 8 cm (Figure 3(a)). Afterwards, an experimenter spread the cuff, placed the LVN into the cuff, and subsequently wetted the LVN with cotton wool dipped into an artificial cerebrospinal fluid (comprising (in mM): MgCl_2_ 2, CaCl_2_ 2, KCl 2.5, NaCl 126, glucose 10, NaH_2_PO_4_·H_2_O 1.25, and NaHCO_3_ 26), oxygenated at room temperature with a mixture of oxygen and carbon dioxide (95% O_2_/5% CO_2_). The cuff was snugly fitted to the wet LVN, covering the entire nerve perimeter, and closed. Finally, the resulting composition was placed into the experimental chamber (Figure 3(b)), which was heated to 37°C using a precision water circulator (Perfectherm PFV, Boehringer, Labor Manheim GmbH für Labortechnik, Germany).

### 2.6. Selective VNS Procedure

LVN was stimulated with a custom-designed stimulator, delivering single stimuli (1 Hz) to the appointed triplet 5. The experiments carried out on each LVN consisted of four chronological subexperiments, referred to as Phases 1–4, where the stimulus preset at the stimulator was recorded simultaneously with the elicited NR. At the beginning of Phases 1–4 the following parameters were fixed: *i*_*c*_ = 4 mA, *t*_*c*_ = 155 *μ*s, *t*_exp⁡_ = 100 *μ*s, *τ*_exp⁡_ = 30 *μ*s, *i*_*a*_ = 0.45 mA, and *t*_*a*_ = 490 *μ*s.

In Phase 1, variable parameter was *i*_*c*_ (range between 0.8 and 4.2 mA); in Phase 2, variable parameter was *t*_*c*_ (range between 60 and 300 *μ*s); and in Phase 3, variable parameter was *τ*_exp⁡_ (range between 20 and to 60 *μ*s); while other parameters remained fixed. In Phase 4, however, variable parameter was the site of NR recording (couple 1–11) (Figure 1(c)) relative to the appointed triplet 5, while stimulating parameters were not of importance because only spatial selectivity was tested in this phase.

Table 3 shows numerical values of the parameters and waveforms that resulted in the most indicative alteration of NR recorded simultaneously in each phase. Regarding the preset stimuli delivered in all four phases to the triplet 5, Table 3 shows the values of the charge *Q*_*c*_ injected in the cathodic phase, as well as values of the charge *Q*_*a*_ injected by a triplet anode in the anodic phase of the stimuli. The triplet anodes and neighboring blocking electrodes were galvanically connected so that the charge density at the anode would be approximately four times lower than that at the cathode. To test the influences of different stimuli on offsets in NR that might be elicited during the selective VNS, the corresponding degrees of imbalance between *Q*_*c*_ and *Q*_*a*_ were calculated and compared.

For the assessment of the population of fibres that most probably contributed to the NR during selective VNS and, consequently, to extract the relevant stimulation and block parameters, patterns recorded in above-mentioned four phases were analyzed.

### 2.7. NR Measurement and Analysis

Unidirectional NR measured with couples 5 or 9 was analysed. The measured NR and voltage drops on the precision serial resistor at the stimulator output were amplified at a differential amplifier (*A* = 100 for the neural response and at *A* = 10 for *i*_*c*_). Afterwards, the data for both signals were gathered at 200 kHz using a high-performance data-acquisition system (DEWE-43, DEWESOFT d. o. o., Republic of Slovenia) and proprietary acquisition software (DEWESoft 7.0.2); the data was then stored on a Lenovo T61 portable computer (Lenovo, Singapore). Offline signal analysis was performed using MATLAB R2007a software (The Mathworks Inc., USA).

To test the paradigm, the components in the NR that had potentially originated from the A- and B-fibres needed to be identified. For this purpose, the integral of the NR observed under the cathodic phase of the stimulus was calculated. This integral actually represented the cumulative contribution of the AP originating from both the A- and B-fibres as well as an ensemble artefact.

To test both, spatial and fibre-type selectivity, the LVN was stimulated with triplet 5, while in Phase 4 the resulting NR were measured from couple 9, actually situated at a site opposite to couple 5 (Figure 1(c)).

In addition, the times considered needed for the AP in the A- and B-fibres to propagate between the different groups of electrodes (Figure 1(d)) are indicated in Table 2. For this purpose, the time separations between the AP of A and B-fibres to reach an inner edge of an anode (Δ*t*_1_), to reach an inner edge of a blocking electrode (Δ*t*_2_), to reach an inner edge of a positive couple electrode (Δ*t*_3_), and to reach a negative couple electrode (Δ*t*_4_) were calculated.

In this stage of the study, the data obtained from the different animals were variable and so could not be effectively combined. Therefore, data from only one animal are presented.

## 3. Results and Discussion

**Figure 4:**
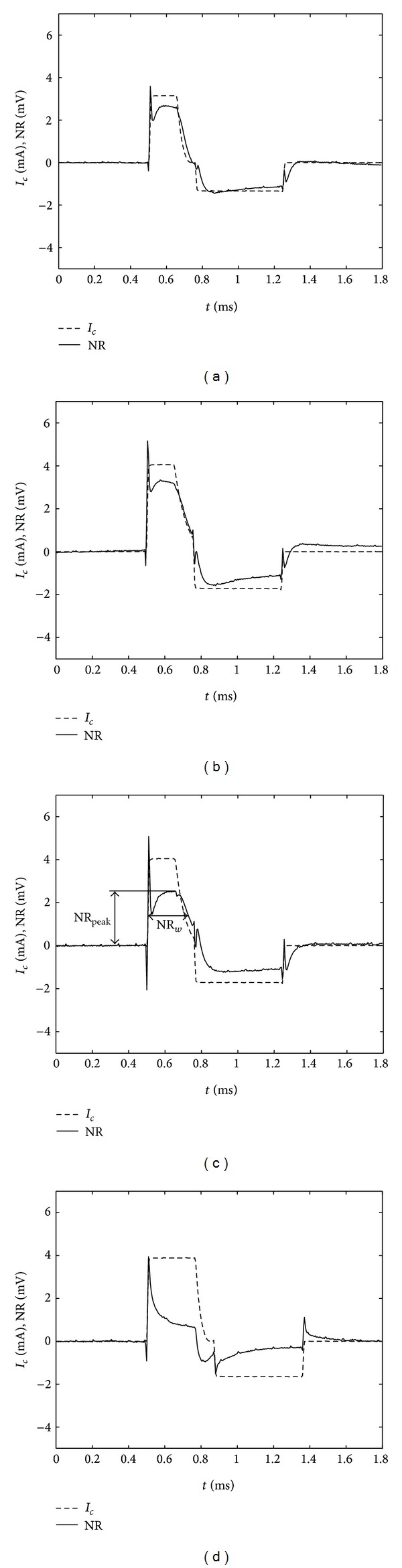
Examples of preset stimulus waveforms and the proprietary NR. Solid line represents stimulating intensity and dashed line represents neural response NR. NR_peak_ is obtained at the top of the bell-shaped NR in the cathodic phase of the stimulus; NR_*w*_ is made at 50% of NR_peak_.

The most indicative alterations obtained in each Phase 1–4, were extracted and presented as the examples of interest (a)–(d). Accordingly, Figure 4 shows examples (a)–(d) comprising preset stimulus waveforms and the proprietary NR.

Example (d), wherein the NR was measured using couple 9, is shown as a reference used for the validity of the paradigm. It was presumed that if the paradigm was valid, then the corresponding NR should not contain the AP of fibres activated with triplet 5. In other words, the integral of the NR manifested under the cathodic phase of the stimulus, representing the cumulative contribution of the AP to the NR, could be interpreted exclusively as an ensemble artefact.

In examples (a)–(c) two components of interest, the amplitude of cathodic peak of the NR (NR_peak_) and the width of the cathodic NR (NR_*w*_), were identified. NR_peak_ was obtained at the top of the bell-shaped NR in the cathodic phase of the stimulus, whereas NR_*w*_ was made at 50% of NR_peak_. With regard to the NR, Table 3 also shows the calculated values of integral 1, manifested under a part of NR belonging to the cathodic phase of the stimulus. These values quantitatively show how the NR changed with different pre-set parameters of the stimuli during selective VNS of the LVN.

From an electrochemical point of view, electrochemical reactions that occured at the cathode owing to the charge *Q*_*c*_ injected via the cathodic phase were reversed only in part by the charge *Q*_*a*_, injected via the anodic phase. Since the triplet anodes and neighboring blocking electrodes were galvanically connected, the charge densities *Q*_*a*_ presented in Table 3 correspond to the charge applied to a single anode within the tested triplet. In examples (a)–(d) (see Figure 4), however, the measured *i*_*a*_ corresponds to the sum of currents delivered to each of four galvanically connected electrodes.

By comparison the examples in Table 3, the largest change in the bell-shaped NR was identified. It was observed in example (a) that *i*_*c*_ influenced both the NR_peak_ as well as NR_*w*_. Precisely, lower *i*_*c*_ elicited slightly lower NR_peak_ and slightly smaller NR_*w*_. Therefore, it is presumed that change of *i*_*c*_ influenced both A- and B-fibres. However, the axons had differing CV and additional numbers of axons firing contributed to the width of the bell-shaped NR, and not only to the peak amplitude. Therefore, NR_*w*_ was indicative of the additional contribution of the B-fibres to the bell-shaped NR when specific parameters and waveforms of the stimulus were used.

It was presumed that the B-fibres contributed the AP that fell towards the region between the apex and the tail section of the bell-shaped NR and that the A-fibres, if not completely blocked, contributed the AP that fell towards the beginning.

It was noticed that parameter *t*_*c*_ did not influence the bell-shaped NR significantly.

One parameter obviously the most important for the confirmation of the paradigm was *τ*_exp⁡_. It could be seen in example (c) when compared to example (b) that larger *τ*_exp⁡_ (45 *μ*s) exhibited significant influence on NR_*w*_. It could be assumed that the AP of the A-fibres was more effectively blocked, whereas the AP of the B-fibres passed through. A certain lack of AP from A-fibres however could also be confirmed by the lower NR_peak_.

It could be noticed that integrals 1 belonging to examples (a) and (c) were significantly smaller compared to integral 1 in example (b). With this regard, it could be speculated that in example (a) less A-fibres were activated, while in example (c) more of them were blocked. It could also be noticed that integral 1 in example (c) is larger when compared to example (a) and is probably a result of easier passage of AP in B-fibres using preset *τ*_exp⁡_ (shown as larger NR_*w*_), while the contribution of A fibres was smaller (shown as lower NR_peak_).

Regarding the offset of the NR, it could be seen that in all four examples (a)–(d) the bell-shaped NR was not greatly influenced by imbalances between *Q*_*c*_ and *Q*_*a*_.

From initial analysis it appeared that the hypothesis stated in the paradigm was shown to be loosely true. Namely, there was a large stimulation artefact superimposed in the NR that greatly obscured the components of the NR, and various components did overlap. These were considered as an ensemble artefact, where the strongest components were thought to arise from the stimulating pulse via the transient response characteristics of an electrode/neural tissue interface and in part from the inherent capacitance of the LVN. An actual ratio between the contribution of the NR and the contribution of the ensemble artefact, relating the waveform of the pulse to the recorded signal, was not defined within this study. In addition, the recordings did not show separate peaks corresponding to the A- and B-fibre types. However, if the distance between the stimulating and recording spiral section was larger, the accuracy of NR measurements would certainly improve [63].

Similar results regarding the selectivity and activation thresholds of the A- and B-fibres were obtained in recent “*in vivo*” studies [64, 65]. Differences in the selective activating and blocking efficacies obtained could be attributed to the different shapes and dimensions of the electrodes used in these studies. If successfully developed further, this paradigm would enable significant improvement of current neuroprostheses. Namely, for neuroprosthetic applications, the usefulness of the paradigm as a framework for determining the activation of combinations of several pathways could certainly be contingent on future developments. Furthermore, a preference towards nerve-fibre type stimulation is advantageous in applications where organ-specific stimulation is required and the side-effect profile related to the propagation of the AP of the A-fibres towards the CNS needs to be minimized. However, more detailed investigations of neural control systems, considering the realistic structural topography of the nerve and the presence of a spatiotemporal constraint based on the electrophysiology of myelinated nerve fibres, should be carried out based on this work. Ultimately, selective NR recording from LVN fibres could be effectively used for closed-loop control of implantable stimulators selectively activating different neural pathways.

## 4. Conclusions

The major finding of the tests carried out on the proposed paradigm, wherein stimulating pulses were applied to preselected locations along an insulated porcine LVN via an appointed stimulating triplet, was the activation of AP in the A- and B-fibres within the corresponding pathways and the slight inhibition of AP in the A-fibres. Activation and inhibition were noticed from the widths and amplitudes of the measured NR to the VNS (bell-shaped NR). Namely, it can be seen in Figure 4 and Table 3 that the bell-shaped NR slightly changed as different parameters and waveforms of the stimulus were chosen. Despite the above-mentioned drawbacks of the paradigm approach used in this study, we achieved an encouraging correlation between the expected and measured NR. In this regard, the directions our further work could take are the following:

- subtraction of the artifacts modifying the components in the NR;
- further development of the cuff;
- testing the ability of the cuff to steer *i*_*c*_ in a desired direction within the LVN;
- testing the ability of the cuff to perform selective VNS in an orthodromic or an antidromic direction in the LVN;
- an independent measurement of compound action potential (CAP) using external hook electrodes;
- comparison of NR measured by the cuff to CAP measured by external hook electrodes;
- performing more experiments to show group statistics.

## Figures and Tables

**Table 1:** Structural topography of the porcine LVN considered in the paradigm.

Diameter of the LVN	*≈*3 mm
Circumference of the LVN	*≈*9 mm
Type of nerve fibres	A, B, and C
Number of B-fibres (%)	300 (60%)
Average area of a single B-fibre	16 *μ*m^2^
Average diameter of a single B-fibre	*≈*4.6 *μ*m
Number of A-fibres (%)	200 (40%)
Number of C-fibres	Majority (not counted)

**Table 2:** Timing of AP propagation in A- and B-fibres between longitudinal electrodes.

Fibre type	*t*_1_ (*μ*s)		*t*_2_ (*μ*s)		*t*_3_ (*μ*s)		*t*_4_ (*μ*s)	
	Max	Min	Max	Min	Max	Min	Max	Min
A	133.3	57.1	266.7	114.3	400.0	171.4	533.3	228.6
B	1333.3	266.7	2666.7	533.3	4000.0	800.0	5333.3	1066.7
Δ*t* (B mean−A mean)	Δ*t*_1_ = 704.8		Δ*t*_2_ = 1992.9		Δ*t*_3_ = 2114.3		Δ*t*_4_ = 3200.0	

*t*_1_: time to reach an inner edge of a triplet anode;

*t*_2_: time to reach an inner edge of a blocking electrode;

*t*_3_: time to reach an inner edge of a positive recording electrode;

*t*_4_: time to reach an inner edge of a negative recording electrode.

**Table 3:** Parameters of the stimuli and values of recorded NR.

Variable	Example (a)	Example (b)	Example (c)	Example (d)
*i*_*c*_ (mA)	3.10	4.00	4.00	4.00
*t*_*c*_ (*μ*s)	155	155	155	265
*t*_exp⁡_ (*μ*s)	100	100	100	100
*t*_au_ (*μ*s)	30	30	45	30
*i*_*a*_ (mA)	−0.4	−0.45	−0.45	−0.45
*t*_*a*_ (*μ*s)	490	490	490	490
*Q*_*c*_ (nAs)	539	770	800	1100
*Q*_*a*_ (nAs)	−163	−208	−208	−200
Integral 1 (nVs)	512.20	665.22	536.22	n/a
NR_peak_ (mV)	2.76	3.17	2.5	n/a
NR_*w*_ (*μ*s)	190	210	222	n/a
